# Supplementary material for: A Likelihood Approach for Real-Time Calibration of Stochastic Compartmental Epidemic Models
Source: PLoS Comput Biol. 2017 Jan 17;13(1):e1005257. doi: 10.1371/journal.pcbi.1005257 (PMC5240920; doi:10.1371/journal.pcbi.1005257)
Supplement: S1 File — (TAR.GZ) [file pcbi.1005257.s014.tar.gz › HSPH_Online-SI-Revision/EnKF07/n100-severe/n100-severe_EnKF_table-4.pdf]

1	R0	{1.65278, 9.06793}	{2.68672, 4.32107}	{{2.15333, 1.27625}, {3., 6.2942}}	{62.5577, 52.3478}	inf	4882.	4080.07	{996.371, 7114.75}	91.3287	0.432903	newly	4385.	9022.38	{1394., 7074.}	198.688	0.429124	Reff	1.41083	0.575296	{0.141368, 0.782102}	69.2764	ar	66 342.	186 624.	{171 305., 222 702.}	181.306
2	R0	{1.4321, 3.80644}	{1.99146, 4.12388}	{{0.0537549, 0.940362}, {3., 5.77864}}	{85.2433, 55.133}	inf	1776.	5183.07	{926.202, 17 614.2}	212.175	0.384705	newly	4438.	5975.	{810., 16 470.}	99.6494	0.385579	Reff	1.34796	0.274429	{0.00755257, 0.455349}	79.6412	ar	54 217.	168 524.	{158 630., 205 301.}	213.524
3	R0	{1.68388, 4.76028}	{2.19776, 4.18807}	{{1.05348, 2.6476}, {3., 4.97095}}	{54.921, 25.2475}	inf	2116.	17 270.7	{5131.68, 57 305.8}	720.835	0.773768	newly	4947.	21 200.4	{5380., 56 459.}	336.324	0.456958	Reff	1.59182	0.42568	{0.0626245, 1.04466}	75.3501	ar	68 299.	170 172.	{98 938., 269 490.}	149.762
4	R0	{1.66014, 6.95383}	{2.47306, 4.33094}	{{1.99662, 1.11115}, {3., 7.41082}}	{53.5773, 42.5317}	inf	3294.	1928.19	{716.575, 2738.79}	76.167	0.482745	newly	4326.	3555.66	{691., 6593.}	95.7665	0.560319	Reff	1.51479	0.576918	{0.175289, 1.89569}	69.0226	ar	66 820.	182 927.	{166 915., 227 968.}	173.76
5	R0	{1.69984, 11.2038}	{2.71918, 4.32944}	{{2.21348, 1.21222}, {3., 6.30072}}	{59.9667, 61.3574}	inf	6557.	3943.71	{901.828, 6088.56}	83.1877	0.510053	newly	4410.	7524.59	{1432., 6781.}	156.702	0.388198	Reff	1.38564	0.57075	{0.12992, 0.769426}	67.7433	ar	68 462.	183 244.	{169 442., 196 067.}	167.657
6	R0	{1.65876, 6.66217}	{2.49925, 4.55174}	{{1.99914, 1.14942}, {3., 7.45541}}	{54.407, 41.1657}	inf	2507.	2015.35	{730.538, 2748.47}	74.9836	0.362612	newly	3575.	3517.21	{691., 6540.}	104.297	0.502246	Reff	1.55026	0.55063	{0.170874, 1.71141}	70.3677	ar	67 105.	182 322.	{166 450., 228 443.}	171.696
7	R0	{1.69966, 5.89996}	{2.02389, 4.3275}	{{0.0624538, 0.93805}, {3., 5.79918}}	{64.6175, 38.9223}	inf	2040.	4547.47	{763.444, 13 345.3}	156.279	0.354215	newly	3565.	5322.64	{594., 9581.}	113.04	0.405098	Reff	1.61145	0.289235	{0.0091387, 0.457253}	82.0512	ar	69 396.	164 369.	{156 655., 185 916.}	142.175
8	R0	{1.53428, 6.3734}	{2.56596, 12.3507}	{{1.26469, 4.87375}, {3., 18.5347}}	{71.9182, 105.057}	inf	2925.	2811.47	{745.909, 2683.14}	53.3778	0.221267	newly	3946.	2704.19	{489., 1875.}	104.459	0.609881	Reff	1.38868	0.56121	{0.254725, 0.945392}	63.9055	ar	59 855.	177 571.	{167 071., 200 712.}	196.668
9	R0	{1.58939, 3.48608}	{2.23059, 4.11629}	{{1.15581, 2.64619}, {3., 5.0012}}	{60.4816, 30.1243}	inf	458.	16 679.	{4951.3, 53 293.4}	3543.75	1.39481	newly	1645.	19 914.1	{4005., 56 356.}	1113.57	0.86521	Reff	1.56577	0.387681	{0.0414861, 0.79067}	76.7577	ar	62 575.	166 881.	{87 472., 265 935.}	168.298
10	R0	{1.58528, 5.5593}	{2.32154, 6.81758}	{{0.929918, 0.5975}, {3., 19.4696}}	{69.5738, 117.479}	inf	2476.	4739.94	{260.713, 6037.25}	128.626	0.481911	newly	4136.	8002.3	{298., 12 454.}	158.636	0.623495	Reff	1.4648	0.504588	{0.145219, 0.670812}	69.0694	ar	63 039.	182 585.	{165 726., 226 299.}	189.639
11	R0	{1.58305, 4.62721}	{1.99442, 4.18973}	{{0.0624737, 0.938204}, {3., 5.79956}}	{72.075, 47.6362}	inf	1827.	5080.26	{818.1, 14 485.9}	203.425	0.387751	newly	3948.	5806.82	{636., 16 485.}	109.525	0.397514	Reff	1.50095	0.280397	{0.00880756, 0.453957}	81.3188	ar	63 222.	166 281.	{155 944., 196 224.}	167.382
12	R0	{1.70234, 9.34626}	{2.70301, 4.28127}	{{2.16039, 1.33475}, {3., 6.29299}}	{58.7821, 54.1927}	inf	5054.	4013.54	{925.815, 7148.45}	89.8252	0.447213	newly	4443.	8514.91	{1415., 7210.}	187.144	0.431907	Reff	1.46651	0.553621	{0.162859, 0.71256}	70.0593	ar	68 213.	185 927.	{169 976., 198 331.}	172.569
13	R0	{1.68556, 9.74954}	{2.68838, 4.2905}	{{2.16329, 1.26725}, {3., 6.29039}}	{59.4946, 55.9928}	inf	5632.	4108.89	{1009.49, 7061.97}	89.2972	0.476421	newly	4572.	9089.4	{1382., 6975.}	192.621	0.430631	Reff	1.41078	0.587983	{0.139111, 0.773413}	68.4016	ar	68 426.	186 610.	{171 305., 223 175.}	172.719
14	R0	{1.59876, 9.78897}	{2.7165, 4.33686}	{{2.17001, 1.27998}, {3., 6.28463}}	{69.913, 55.6965}	inf	4188.	3181.77	{796.18, 5773.}	75.3561	0.386118	newly	3466.	6960.23	{1321., 6995.}	181.294	0.368287	Reff	1.39393	0.54469	{0.141506, 0.774773}	72.0435	ar	63 553.	184 628.	{170 303., 197 047.}	190.511
15	R0	{1.43577, 5.12819}	{2.23665, 5.79682}	{{0.111847, 3.46488}, {3., 12.5042}}	{83.7855, 47.3717}	inf	1915.	7830.98	{141.931, 4296.28}	349.136	0.573942	newly	3233.	6269.52	{158., 6354.}	147.075	0.462582	Reff	1.33374	0.495358	{0.0303563, 0.712357}	64.0352	ar	53 259.	183 745.	{166 661., 177 877.}	245.003
16	R0	{1.58866, 8.73191}	{2.39092, 6.9962}	{{0.932393, 0.602234}, {3., 19.3765}}	{71.9635, 93.0133}	inf	3460.	4983.57	{476.598, 5967.29}	79.6135	0.341559	newly	3246.	8303.88	{452., 13 301.}	211.486	0.562056	Reff	1.43615	0.536822	{0.176466, 0.67668}	66.9365	ar	64 349.	182 882.	{166 376., 224 267.}	184.203
17	R0	{1.43012, 3.8163}	{2.10548, 4.11378}	{{0.0700133, 0.942847}, {3., 5.7977}}	{85.2702, 61.2073}	inf	970.	5103.65	{741.631, 13 984.}	436.049	0.543002	newly	2605.	6687.17	{576., 15 452.}	204.656	0.435575	Reff	1.37832	0.341495	{0.0105773, 0.660056}	75.4807	ar	53 353.	164 318.	{152 947., 196 759.}	212.963
18	R0	{1.47965, 5.16472}	{2.49633, 6.51938}	{{1.43355, 3.28808}, {3., 10.0939}}	{69.9537, 51.8985}	inf	2352.	2101.24	{1450.12, 1954.59}	63.7161	0.324579	newly	4058.	3031.49	{698., 3270.}	100.158	0.620587	Reff	1.36212	0.47694	{0.0336319, 0.948775}	66.0422	ar	56 086.	179 091.	{168 110., 212 045.}	219.316
19	R0	{1.57191, 5.2314}	{2.47888, 6.57793}	{{1.42183, 3.24271}, {3., 10.0217}}	{61.6927, 51.0162}	inf	3247.	2153.71	{61.2597, 2094.36}	86.6842	0.715046	newly	5657.	2952.88	{28., 2603.}	99.8784	1.0263	Reff	1.41239	0.46867	{0.0262309, 0.943449}	67.1281	ar	63 119.	179 079.	{166 804., 211 001.}	183.716
20	R0	{1.54435, 5.32911}	{2.57287, 12.3056}	{{1.29978, 4.87967}, {3., 18.6138}}	{70.8143, 134.94}	inf	2562.	2618.06	{460.944, 2739.2}	58.2132	0.251748	newly	4511.	2602.1	{397., 1897.}	103.388	0.723	Reff	1.42398	0.540738	{0.22945, 0.717727}	65.9028	ar	61 870.	176 330.	{166 214., 173 782.}	185.001
21	R0	{1.57932, 4.90031}	{2.02403, 4.34506}	{{0.063924, 0.939312}, {3., 5.80002}}	{72.2878, 46.0801}	inf	1638.	4687.67	{765.602, 14 323.9}	210.33	0.388871	newly	3321.	5457.07	{609., 13 646.}	124.618	0.407715	Reff	1.50468	0.28879	{0.00919293, 0.457823}	80.8072	ar	63 595.	164 581.	{156 054., 189 311.}	163.607
22	R0	{1.47044, 4.07694}	{2.50248, 6.68065}	{{1.45179, 3.34461}, {3., 10.2073}}	{70.6616, 75.8608}	inf	941.	2982.76	{1488.83, 4391.38}	218.095	0.305045	newly	2369.	3944.49	{744., 3662.}	144.966	0.366204	Reff	1.4165	0.517842	{0.0235149, 0.9757}	65.5783	ar	55 895.	181 781.	{170 229., 216 248.}	225.218
23	R0	{1.64997, 3.34546}	{2.4507, 4.8339}	{{1.76642, 1.19044}, {3., 12.757}}	{49.3896, 94.2135}	inf	758.	20 380.3	{4139.28, 54 048.9}	2591.21	1.24964	newly	3196.	25 440.1	{2380., 72 868.}	707.807	0.731907	Reff	1.61331	0.652958	{0.168375, 1.36246}	61.314	ar	66 404.	154 975.	{97 275., 247 726.}	134.542
24	R0	{1.67405, 4.50254}	{2.52449, 7.28482}	{{2.16876, 4.22619}, {3., 14.6897}}	{52.9191, 69.8522}	inf	1073.	22 159.9	{7627.77, 42 885.4}	1965.23	1.18373	newly	2825.	23 231.7	{6140., 53 237.}	725.252	0.77422	Reff	1.63156	1.00549	{0.309018, 2.18533}	52.7631	ar	68 017.	153 209.	{93 686., 222 407.}	126.713
25	R0	{1.50663, 3.1286}	{2.52415, 7.32995}	{{2.16878, 4.23555}, {3., 14.6932}}	{69.1947, 136.879}	inf	639.	22 100.1	{7642.29, 42 801.5}	3358.55	1.40692	newly	2373.	23 121.3	{6093., 53 227.}	876.138	0.839536	Reff	1.47669	1.00563	{0.309117, 2.18492}	52.4228	ar	58 823.	152 817.	{85 535., 222 444.}	160.88
26	R0	{1.69822, 6.34992}	{2.18801, 8.59397}	{{0.371783, 2.65606}, {3., 13.5893}}	{66.3144, 66.7396}	inf	2762.	4345.36	{351.037, 2783.82}	97.5038	0.309545	newly	4173.	3632.29	{165., 4221.}	87.0201	0.578338	Reff	1.57542	0.40459	{0.0696034, 0.652858}	74.4842	ar	69 816.	177 541.	{168 708., 194 559.}	154.298
27	R0	{1.55869, 4.92988}	{2.48495, 6.57801}	{{1.44012, 3.30171}, {3., 10.1585}}	{62.6104, 53.4648}	inf	1917.	2172.72	{1474.86, 2103.43}	58.8574	0.223196	newly	3834.	3109.65	{717., 3328.}	100.273	0.573523	Reff	1.46882	0.489395	{0.0284897, 0.956912}	66.8532	ar	62 344.	179 162.	{167 992., 212 955.}	187.377
28	R0	{1.46085, 6.06899}	{2.14785, 8.50861}	{{0.333616, 2.58479}, {3., 13.3474}}	{85.5566, 71.1715}	inf	3200.	3578.96	{154.551, 2684.2}	104.922	0.572993	newly	4410.	2878.36	{113., 3999.}	96.9764	0.812402	Reff	1.29135	0.411917	{0.0662065, 0.635727}	68.6333	ar	55 484.	176 099.	{166 788., 190 654.}	217.387
29	R0	{1.63017, 6.25164}	{2.48308, 6.60521}	{{1.42911, 3.26603}, {3., 10.0612}}	{57.4867, 45.4133}	inf	3520.	1899.65	{66.0076, 1989.68}	72.9377	0.656754	newly	5090.	2724.26	{35., 2262.}	95.5886	0.903352	Reff	1.47729	0.475094	{0.0375539, 0.723136}	67.9883	ar	66 231.	178 123.	{166 754., 200 976.}	168.943
30	R0	{1.45173, 5.68165}	{2.36898, 6.55776}	{{0.929549, 0.622958}, {3., 19.4393}}	{81.8642, 115.317}	inf	2741.	4821.06	{458.009, 5951.41}	107.519	0.392021	newly	3975.	8156.99	{485., 12 046.}	164.869	0.550654	Reff	1.31862	0.494716	{0.139278, 0.622983}	67.2394	ar	55 280.	182 403.	{166 351., 226 438.}	229.962
31	R0	{1.55808, 2.77316}	{2.5323, 7.28913}	{{2.16542, 4.23228}, {3., 14.6899}}	{64.3371, 164.491}	inf	583.	22 020.1	{5948.94, 42 845.6}	3677.03	1.43914	newly	3043.	23 090.9	{6129., 53 281.}	662.715	0.73936	Reff	1.52792	1.00555	{0.30947, 2.1873}	52.5893	ar	60 940.	153 093.	{85 678., 222 283.}	152.419
32	R0	{1.56797, 3.90327}	{2.28257, 4.18001}	{{1.15133, 2.64665}, {3., 4.9893}}	{64.3953, 28.08}	inf	984.	13 601.3	{5059.24, 24 181.8}	1286.11	1.03353	newly	2775.	15 960.6	{4345., 41 558.}	479.819	0.62429	Reff	1.52355	0.408642	{0.0512036, 0.893381}	73.2221	ar	62 228.	160 391.	{95 769., 203 369.}	158.486
33	R0	{1.70027, 5.43261}	{2.19722, 4.18058}	{{1.05479, 2.6481}, {3., 4.95636}}	{54.134, 30.287}	inf	2721.	17 273.1	{5094.29, 57 209.5}	539.806	0.670543	newly	5236.	21 217.3	{5294., 56 339.}	312.917	0.433421	Reff	1.58887	0.42555	{0.0625673, 1.04577}	75.32	ar	68 798.	169 864.	{99 710., 269 406.}	147.469
34	R0	{1.61668, 6.86192}	{2.64227, 4.05772}	{{2.17847, 1.39252}, {3., 6.30187}}	{63.4383, 40.8661}	inf	3615.	5811.28	{714.985, 8838.87}	138.123	0.396634	newly	4645.	14 193.7	{1210., 32 223.}	298.602	0.52072	Reff	1.44922	0.620986	{0.162394, 1.92429}	70.2679	ar	64 693.	195 323.	{171 601., 268 492.}	201.923
35	R0	{1.55955, 3.45071}	{2.37422, 4.66212}	{{1.76456, 1.19543}, {3., 12.7693}}	{53.034, 87.5466}	inf	643.	23 706.7	{4852.83, 54 029.5}	3588.83	1.39189	newly	2408.	29 848.3	{2471., 73 689.}	1144.88	0.915052	Reff	1.53037	0.725384	{0.252046, 1.36339}	56.3736	ar	61 783.	162 357.	{102 773., 252 870.}	163.734
36	R0	{1.65068, 4.91307}	{2.02814, 4.07896}	{{0.0759335, 0.936178}, {3., 5.81533}}	{67.3493, 46.1351}	inf	1136.	5454.96	{760.929, 14 377.2}	391.998	0.515922	newly	2506.	6951.74	{572., 16 680.}	220.688	0.431795	Reff	1.60096	0.327361	{0.0110913, 0.523621}	79.5522	ar	66 453.	168 775.	{156 399., 227 245.}	159.754
37	R0	{1.46131, 3.06714}	{2.4697, 4.72022}	{{1.81112, 1.16073}, {3., 12.7279}}	{69.7011, 106.691}	inf	856.	21 491.7	{4195.01, 54 167.1}	2411.92	1.2237	newly	3280.	26 822.9	{2454., 72 799.}	728.017	0.739397	Reff	1.41952	0.637361	{0.162525, 1.27378}	57.946	ar	56 452.	152 973.	{97 628., 246 859.}	171.09
38	R0	{1.71034, 5.2546}	{1.99488, 4.19393}	{{0.0631845, 0.933294}, {3., 5.8017}}	{64.1966, 41.4355}	inf	2107.	5063.41	{785.456, 14 458.7}	172.411	0.370175	newly	4350.	5805.63	{616., 16 589.}	101.673	0.41071	Reff	1.62197	0.28274	{0.00895582, 0.45271}	82.5681	ar	69 816.	166 850.	{156 412., 196 933.}	144.429
39	R0	{1.43565, 5.57989}	{2.15164, 8.52993}	{{0.341941, 2.60212}, {3., 13.3772}}	{87.9149, 81.6589}	inf	2976.	3662.5	{141.583, 2700.14}	104.436	0.555947	newly	4327.	2928.23	{102., 4126.}	96.8687	0.811647	Reff	1.28285	0.412557	{0.0677145, 0.635506}	68.4102	ar	54 407.	176 160.	{166 419., 189 578.}	223.781
40	R0	{1.40327, 4.00764}	{1.99231, 4.17682}	{{0.0577532, 0.947722}, {3., 5.76334}}	{87.9893, 55.0151}	inf	1552.	5096.46	{845.711, 14 497.7}	247.066	0.417014	newly	3528.	5803.12	{662., 16 424.}	120.529	0.387122	Reff	1.32289	0.277372	{0.00811976, 0.453828}	79.0329	ar	51 976.	166 669.	{157 816., 196 320.}	223.594
41	R0	{1.63612, 4.26741}	{2.45865, 4.5548}	{{1.80558, 1.1068}, {3., 12.6965}}	{50.2731, 75.2961}	inf	1646.	22 514.8	{4880.17, 58 066.}	1270.14	0.976635	newly	4452.	27 160.8	{2619., 72 182.}	521.624	0.647413	Reff	1.56264	0.597687	{0.124249, 1.0365}	63.0111	ar	65 029.	154 744.	{98 083., 247 287.}	138.361
42	R0	{1.57426, 6.50271}	{2.23285, 5.60519}	{{0.0914809, 3.46642}, {3., 12.4954}}	{70.3989, 48.8216}	inf	3056.	6824.63	{360.451, 4312.63}	176.875	0.442031	newly	4138.	5732.98	{344., 6348.}	110.073	0.446735	Reff	1.43171	0.495729	{0.025642, 0.714184}	66.0825	ar	62 522.	181 641.	{166 910., 177 858.}	190.523
43	R0	{1.37818, 5.37636}	{2.36805, 7.0153}	{{0.923103, 0.643362}, {3., 19.3893}}	{89.6303, 125.861}	inf	2468.	4872.51	{614.696, 5863.67}	120.99	0.384697	newly	3812.	7782.35	{593., 11 927.}	162.339	0.52434	Reff	1.24355	0.521518	{0.138772, 0.688946}	64.2877	ar	50 098.	182 654.	{166 430., 226 279.}	264.594
44	R0	{1.66606, 4.92641}	{2.28255, 4.164}	{{1.15138, 2.64673}, {3., 4.98743}}	{58.4798, 22.0979}	inf	1205.	13 599.7	{5051.64, 24 167.9}	1032.96	0.953429	newly	2554.	15 971.7	{4363., 41 559.}	529.747	0.660951	Reff	1.61485	0.41025	{0.0639748, 0.892556}	74.5951	ar	67 066.	160 563.	{97 231., 202 475.}	140.497
45	R0	{1.623, 5.9449}	{2.54152, 4.46437}	{{1.99973, 1.17458}, {3., 7.44933}}	{59.5327, 44.61}	inf	2171.	1994.41	{728.177, 2770.13}	76.2152	0.332879	newly	3513.	3424.79	{681., 6517.}	104.839	0.516272	Reff	1.52557	0.522186	{0.171119, 1.04245}	71.2405	ar	64 554.	180 657.	{166 267., 218 954.}	179.854
46	R0	{1.65235, 5.51234}	{2.47576, 6.62105}	{{1.44795, 3.32509}, {3., 10.2029}}	{55.0229, 51.2572}	inf	1774.	2281.54	{1481.34, 2915.98}	57.2436	0.140622	newly	3211.	3180.93	{748., 3558.}	104.162	0.450535	Reff	1.57561	0.476977	{0.0237593, 0.727181}	69.7514	ar	66 509.	179 153.	{169 569., 201 120.}	169.366
47	R0	{1.62138, 8.82208}	{2.33822, 6.98475}	{{0.627791, 0.735258}, {3., 19.3064}}	{76.0415, 92.1202}	inf	5092.	5670.46	{893.662, 6234.36}	49.5441	0.237709	newly	4726.	8209.17	{822., 13 443.}	133.494	0.465526	Reff	1.36283	0.516162	{0.0820454, 0.726418}	66.2797	ar	65 598.	182 079.	{166 949., 216 514.}	177.568
48	R0	{1.64073, 6.69261}	{2.48099, 4.43302}	{{1.99718, 1.12872}, {3., 7.41885}}	{55.6655, 41.9589}	inf	3000.	1997.69	{719.077, 2678.05}	76.5537	0.457213	newly	4095.	3513.08	{708., 6462.}	96.4415	0.559397	Reff	1.5074	0.584738	{0.177144, 1.99716}	69.0819	ar	66 438.	183 256.	{166 555., 246 882.}	175.83
49	R0	{1.65556, 3.796}	{2.56282, 7.04537}	{{2.16248, 4.26459}, {3., 14.6911}}	{57.497, 89.5902}	inf	673.	22 274.	{6633.57, 38 404.9}	3209.65	1.38342	newly	2370.	24 100.5	{6122., 66 635.}	920.043	0.85118	Reff	1.6296	0.937983	{0.325329, 2.18643}	54.196	ar	66 575.	150 133.	{85 294., 222 267.}	127.912
50	R0	{1.56828, 3.65713}	{2.47438, 4.54818}	{{1.81038, 1.11058}, {3., 12.707}}	{57.7766, 87.4776}	inf	1346.	22 368.9	{4372.65, 58 125.8}	1564.73	1.04897	newly	4132.	26 953.	{2403., 72 090.}	565.434	0.672262	Reff	1.50552	0.592805	{0.12634, 1.04263}	62.0802	ar	62 761.	154 527.	{97 937., 247 088.}	146.529
